# Prominent role of RAB39A-RXRB axis in cancer development and stemness

**DOI:** 10.18632/oncotarget.23955

**Published:** 2018-01-04

**Authors:** Tokuhiro Chano, Hiroko Kita, Sofia Avnet, Silvia Lemma, Nicola Baldini

**Affiliations:** ^1^ Department of Clinical Laboratory Medicine, Shiga University of Medical Science, Otsu, Shiga, Japan; ^2^ Orthopaedic Pathophysiology and Regenerative Medicine Unit, Istituto Ortopedico Rizzoli, Bologna, Italy; ^3^ Department of Biomedical and Neuromotor Sciences, University of Bologna, Bologna, Italy

**Keywords:** RAB39A, RXRB, cancer stem cell, spherogenicity, tumorigenesis

## Abstract

In this study, we found that *RAB39A*, a member of the RAS oncogene family, was selectively expressed in cancer cells of different histotypes, by analyzing gene expression in human osteosarcoma cells and the cancer stem cells (CSCs) and by comparing them with normal cells through global transcriptomics and principal component analyses. We further validated *RAB39A* as a therapeutic target, by silencing its expression. The silencing impaired cancer stemness and spherogenic ability *in vitro*, as well as tumorigenesis *in vivo*. RNA-seq analyses in the silenced spheres suggested that RAB39A is associated downstream with RXRB and KLF4. Notably, *RXRB* expression was inhibited in *RAB39A*-silenced CSCs. Induced overexpression of *RXRB* in *RAB39A*-silenced cells restored spherogenic ability and tumorigenesis, confirming RXRB as a major effector of RAB39A. Quantitative RT-PCR analysis of ∼400 human cancer tissues showed that *RAB39A* was highly expressed in sarcomas and in malignancies of lymphoid, adrenal and testicular tissues. Our data provide the rationale for targeting of the RAB39A-RXRB axis as a therapy for aggressive cancers.

## INTRODUCTION

Cancers develop in a variety of microenvironmental conditions and under the selective pressure of hostile conditions, such as low extracellular pH, low oxygen levels, and low metabolite concentrations [1]. Such hostile microenvironments influence tumor growth, progression, invasiveness, immune escape, and drug resistance [2, 3]. Studies aimed at identifying anticancer molecules need to take into account the tumor microenvironment and its features. An important aspect of the tumor microenvironment is cancer-associated extracellular acidosis, which is a general phenomenon mainly derived from the metabolic switch to persistent aerobic glycolysis, even under adequate oxygen conditions, the so called “Warburg effect” [4]. Acidosis plays a critical role in the progression of many cancers as it fosters chemo- and radioresistance, neo-angiogenesis, invasion and stemness [5]. On this basis, we used various “-omics” approaches to identify specific and reliable anticancer therapeutic targets [3, 6, 7] by comparing gene expression patterns in cancer cells and normal cells. The normal cells used here as a reference were fibroblasts (Fb) and mesenchymal stem cells (MSCs), cultured under both acidic and neutral environments. The information gathered in this comparative study should provide the basis for the development of novel and selective anticancer therapeutics.

The present study shows that RAB39A (RAB39A, Member of the RAS oncogene family) is a novel therapeutic target and that RXRB (Retinoid X Receptor Beta) is one of its major effectors. RAB39A is associated with the acidification of phagosomes during the maturation phase into lysosomes [8, 9]. RXRB is a member of the retinoid X receptor (RXR) families mediating the effects of retinoic acid [10], and the mutual heterodimers with vitamin D receptor (VDR) are involved in cancer developments [11]. Even though RAB39A-RXRB axis has never been described as a therapeutic option for cancers, here, it is proved that targeting RAB39A-RXRB axis significantly impairs cancer stem cell (CSC) growth and survival. Thus, targeting RAB39A-RXRB axis is a promising therapeutic approach to specifically affect the CSC fraction of the total cancer cell population that is also the tumor cell fraction associated with chemoresistance, recurrence, and metastasis.

## RESULTS

### RAB39A is a novel target for molecular anticancer therapeutic strategies

As a first step, we compared global transcription differences of cancer cells and CSCs with normal cells cultured under acidic pH conditions versus neutral conditions. CSCs were enriched by sphere-forming culture, the most widely accepted method for isolating CSCs [12]. Under acidosis, we found that both cancer cells and CSCs showed up-regulation of pathways involved in DNA replication and cell proliferation (Supplementary Table 1). In contrast, Fb and MSCs that were maintained at low pH switched to inflammatory pathways (Supplementary Table 2). According to our previous data, this switch in Fb and MSCs may enhance invasiveness and metastatic potential of cancer cells, including CSCs [7, 13].

**Figure 1:**
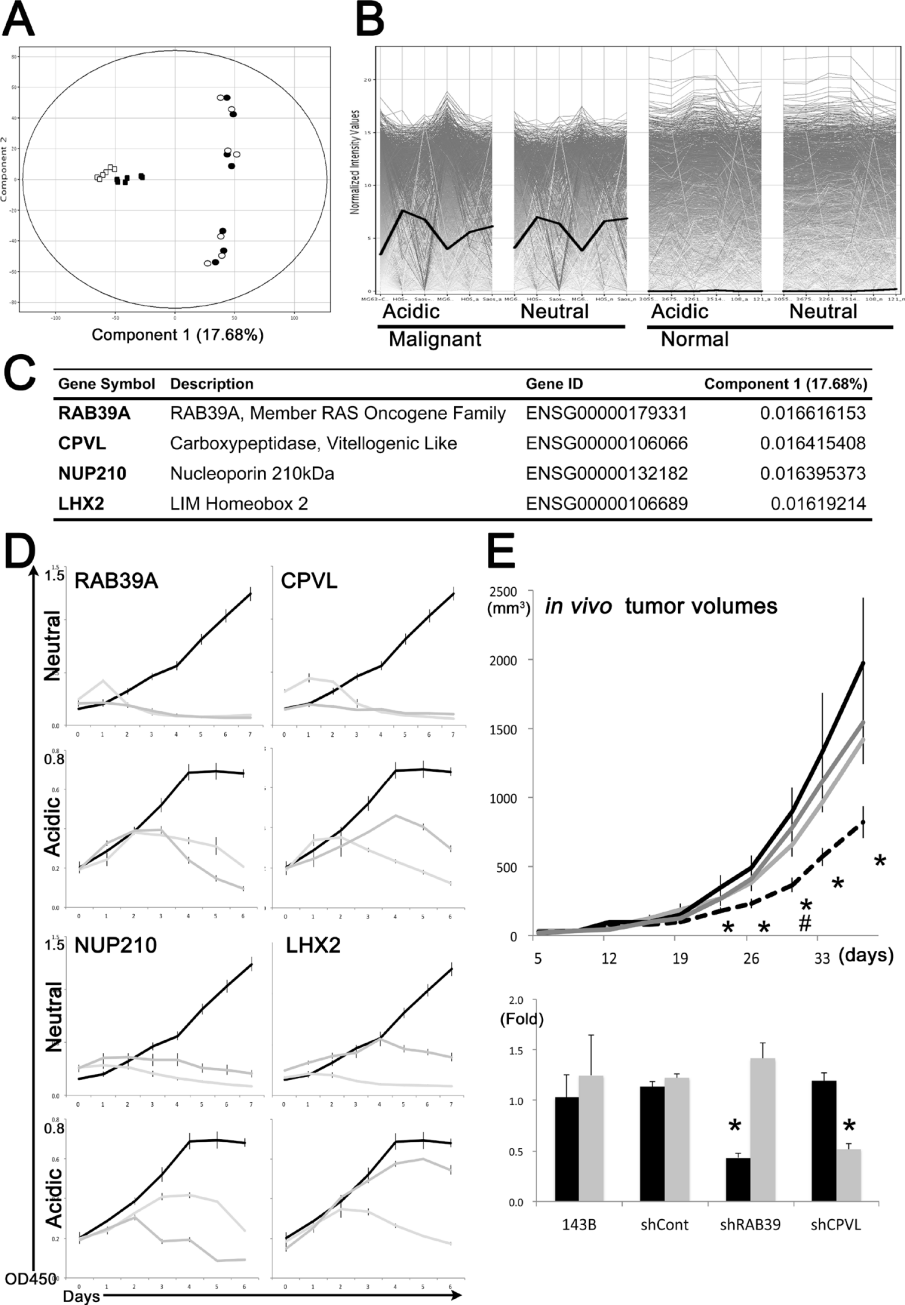
Transcriptional comparison, mathematical ranking and shRNA-based approaches for selection of candidate genes with the best ranking for cancer targeting (**A**) Sarcoma cells were distinguished from normal cells by component 1 axis of a principal component analysis, followed by global transcriptome comparison. White and black indicate transcriptional status under neutral (pH 7.4) and acidic (pH 6.5) conditions, respectively. Round and square symbols indicate sarcoma and normal cells, respectively. (**B**) *RAB39A* (bold black line) shows higher expression values in malignant cells. Each line indicates the expression value of a gene. Genes expressed at low levels in normal cells but abundantly expressed in malignant cells were identified as targeting candidates, and used for shRNA-based screening. (**C**) *RAB39A*, *CPVL*, *NUP210* and *LHX2* are primary candidates for targeting cancer cells and CSCs. (**D**) Cell growth inhibition in neutral (pH 7.4) and acidic (pH 6.8) conditions in cells transfected with shRNAs against the selected genes. Black and 2 gray lines indicate growth of shControl and 2 kinds of gene-specific shRNA-treated HOS human osteosarcoma cells, respectively. (**E**) Upper graph, reduced tumor volumes in mice subcutaneously injected with *RAB39A*-knockdown 143B human osteosarcoma cells (black dotted line). Black, dark gray and light gray lines correspond to shControl, shCPVL and parental 143B cells, respectively. #: ANOVA on Day 30; F (3, 20) = 4.054, *p* = 0.0211 ^*^p < 0.05, by Fisher’s PLSD, compared to shControl cells. Lower graph, silencing of *RAB39A* and *CPVL* expression (black and gray bars, respectively) in 143B xenotransplanted tumors with different cell lines was confirmed by qRT-PCR.

To identify novel molecular targets for the development of anticancer therapies, we performed a principal component analysis (PCA) on the global transcriptome data. Cancer cells, including CSCs differed from normal cells in the component 1 axis of PCA (Figure 1A). Among the top 100 gene elements on this axis (Supplementary Table 3), we selected 10 candidates, including *RAB39A*, *CPVL*, *NUP210* and *LHX2,* which were robustly expressed in cancer cells and CSCs but not in normal MSCs or Fb in both acidic and neutral environments (Figure 1B).

ShRNA-based stable knockdown of *RAB39A*, *CPVL*, *NUP210* and *LHX2* significantly impaired cancer cell growth under neutral and acidic conditions in HOS human osteosarcoma cells (Figure 1C and 1D), but had no effect on the growth of normal cells (Supplementary Figure 1A). As a result of this experiment, we considered these 4 candidate genes as promising therapeutic targets for cancer. We also identified four other genes from the component 1 axis as second-choice targets (Supplementary Figure 1B), namely, *KCNG3*, *SLITRK5*, *FXYD6* and *PRAME*. Silencing of these latter genes inhibited cell growth in neutral conditions but not in acidic conditions (Supplementary Figure 1C). Similar effect was confirmed in HeLa human endocervical carcinoma cells (data not shown).

The stable knockdown of *RAB39A* in 143B human osteosarcoma cells, which originated from the same host of HOS cells and which are well-known to form xeno-transplanted tumors in immunodeficient mice, resulted in a significantly lower rate of tumorigenicity following xenotransplantation into a mouse model compared to shControl-143B and the parental cells. Silencing of *CPVL* couldn’t significantly affect *in vivo* tumor growth (Figure 1E). The differences in rates of tumor development might result from the transplantability of each type of knockdown cell, and correspond to the variable survival rate of each type of knockdown cell at the initial stages of xenotransplantation, i.e., the poor tumorigenicity of *RAB39A*-knockdown cell was possibly related to the reduced cancer stemness in the cell. Indeed, cancer stemness is a phenotype that is associated with the ability to reproduce a similar tumor to the original one in immunodeficient mouse models, even after several passages in the cell culture [12, 14].

### Targeting RAB39A inhibits cancer stemness and tumorigenesis

**Figure 2:**
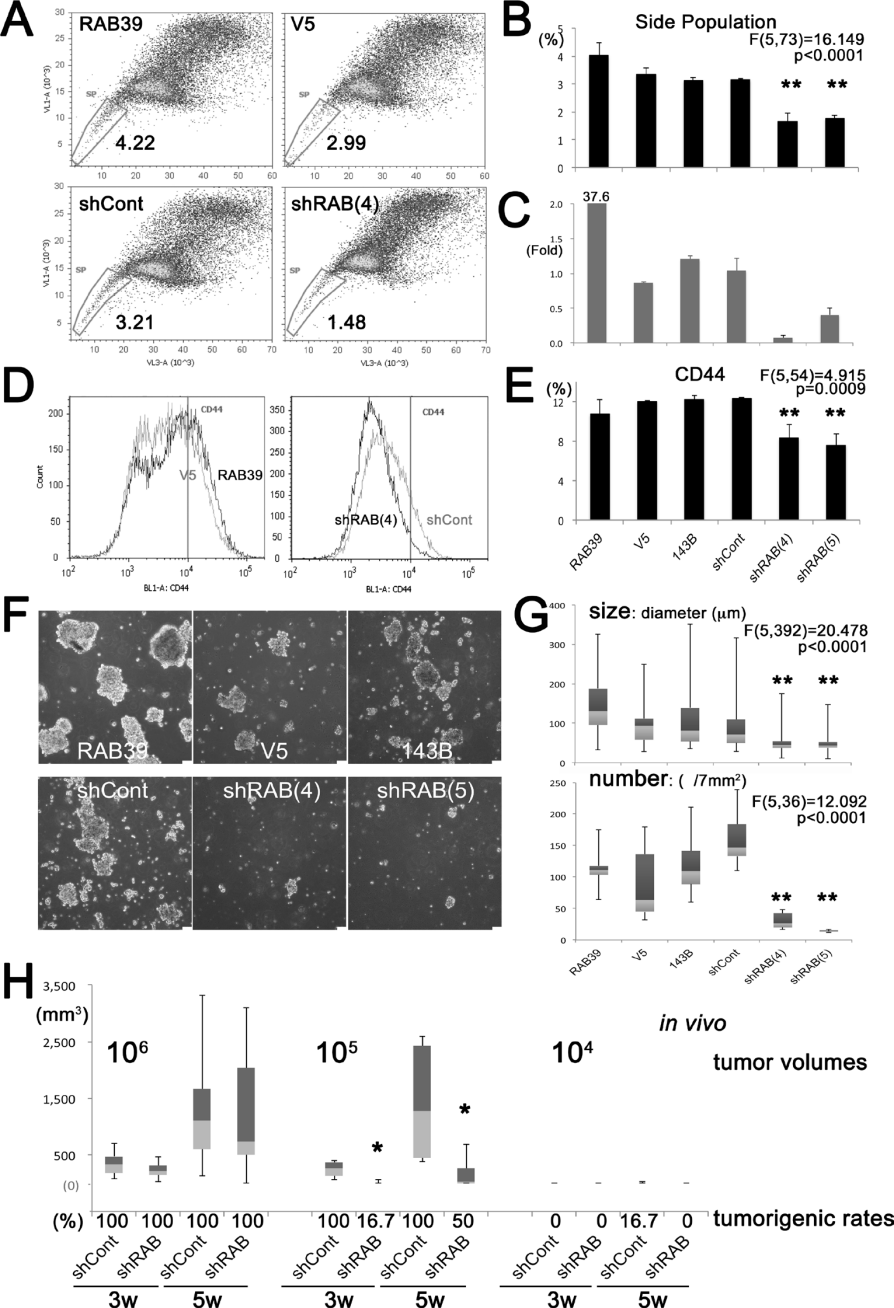
Knockdown of RAB39A reduces stemness and tumorigenicity All experiments were performed using 143B cells. (**A**) Flow cytometry of SP fraction of cells transfected with different vectors: RAB39, cells transfected with *RAB39A*; control cells (V5), cells transfected with *RAB39A* RNAi (shRAB(4)) or with control RNAi (shCont). (**B**) Graphic representation of data shown in panel A. In *RAB39A* silenced cells, SP fractions were significantly reduced. (**C**) *RAB39A* expression was confirmed by qRT-PCR. *RAB39A* knockdown used either shRAB (4) or shRAB (5). (**D**) Flow cytometry of CD44 expression. Left panel, RAB39 (black line) and V5 (gray line); right panel, shRAB (4) (black line) and shCont (gray line). (**E**) Graphic representation of data shown in panel D. Knockdown of *RAB39A* significantly inhibited CD44 v9 expression. (**F**) Reduced rate of sphere formation after knockdown of *RAB39A*. Scale bar, 75 µm. (**G**) After knockdown of *RAB39A*, sphere size (diameter) and number were significantly reduced. ^**^p < 0.05, by Fisher’s PLSD, vs. shCont and parental 143B cells. (**H**) Six mice were xenotransplanted by subcutaneous injection with serial dilutions (10^6^–10^4^) of tumor cells transfected with different vectors. With 10^5^-dilution, tumor volumes were significantly smaller than *RAB39A* knockdown tumors (shRAB) at 3 (*p* = 0.0048) and 5 weeks (*p* = 0.0156, Mann-Whitney *U*-test vs. shCont). Rates (%) of tumorigenesis with respect to control tumors (shCont) are shown. shRAB and shCont correspond to shRAB (4) and shCont in the *in vitro* experiments.

According to our previous results, sarcoma CSCs show a higher level of lysosomal acidity [15]. RAB39A is associated with the acidification of phagosomes during the maturation phase into lysosomes [8, 9], and we thus speculated that CSCs are a good target for anti-RAB39A strategies. Here, we sought to confirm the effectiveness of RAB39A targeting for the CSC fraction of a tumor cell population, by knocking-down or over-expressing *RAB39A* in parental 143B and HeLa cells via lentiviral transduction with specific vectors. We evaluated the proportion of the side population (SP) within the total tumor cell population. In 143B human osteosarcoma cells, the overexpression of *RAB39A* increased the SP fraction whereas the knockdown of *RAB39A* significantly decreased the SP fraction (Figure 2A and 2B). Induced overexpression or knockdown of *RAB39A* was confirmed by quantitative reverse transcription polymerase chain reaction (qRT-PCR) analysis (Figure 2C). *RAB39A* knockdown in 143B tumor cell population also impaired the expression of the stem cell marker CD44 compared to control (shCont) cells (Figure 2D and 2E). The results obtained with osteosarcoma cells were confirmed for HeLa cells in which we found a higher expression of the CXCR4 stem cell marker, a higher SP fraction in cells overexpressing *RAB39A*, and a reduced CXCR4 expression and SP fraction in cells with knockdown of *RAB39A* (Supplementary Figure 2A to 2F). As an index of stemness, we also evaluated sphere-forming ability. This assay is the most widely accepted method for isolating CSCs [12] and is based on their capacity to grow as floating spheres in the absence of fetal serum. Sphere formation was significantly reduced after treatment with 2 types of *RAB39A* knockdown, and silencing with either shRAB (4) or (5) (Figure 2F, Supplementary Figure 2G) significantly affected the number and the size of the obtained spheres (Figure 2G, Supplementary Figure 2H).

In order to evaluate the impact of RAB39A on tumorigenesis, *RAB39A*-knockdown (shRAB) cells and control 143B (shCont) cells were injected subcutaneously at serially different cell dilutions into the left and right sides, respectively, of the backs of mice. After xenotransplantation, tumor volumes and tumorigenic rates were measured at 3 and 5 weeks. We observed a significantly lower rate of tumorigenicity in the left sides of mice with transplantation of *RAB39A*-knockdown cells at a 10^5^ cell dilution at both time points (Figure 2H, Supplementary Figure 3A).

### RXRB is the downstream effector for RAB39A-mediated induction of cancer stemness

In order to identify the downstream pathways of RAB39A, which ultimately affect cancer stemness, we used RNA-seq to screen the RNA profile of spheres formed by 143B and HeLa cells with knockdown of *RAB39A.* We compared transcription patterns in spheres obtained by silencing *RAB39A* (shRAB (4) and (5)) with spheres from parental cells, spheres from *RAB39A*-overexpressing cells (RAB39), and with control cells (shCont and V5, which are shRABs and RAB39 controls, respectively). We used stringent criteria to select candidate genes that showed at least 2-fold greater expression and were statistically replicated in spheres of *RAB39A* knockdown cells; the fold differences and statistical significance were confirmed in both 143B and HeLa cells (Table 1). The identified genes were processed using the “Pathway Analysis” software package (Strand NGS software version 2.0), and we focused on down-regulated and up-regulated pathways (Table 2). “Notch Signaling”, “Transcriptional activity of SMAD2-3-4 heterotrimer” and “Aryl Hydrocarbon Receptor” were identified as down-regulated pathways that are involved in NCOR2 inhibition. “Vitamin D Metabolism”, “Nuclear Receptors”, “Signaling by Retinoic Acid” and “Vitamin A and Carotenoid Metabolism” were identified as down-regulated pathways that are involved in RXRB (Retinoid X Receptor Beta) inhibition. UGT2B15 and RIIAD1 were involved in the most important up-regulated pathways.

**Table 1:** Genes that were significantly changed in RAB39A-silenced and slowly growing spheres

Down_Gene Symbol	Gene ID	Fold Change^*^	*p*-values (Corr) ^**^
MIR3916	100500849	-4.2365184	0
JMY	133746	-2.0020356	0
AMH	268	-5.758692	0
SNORD35B	84546	-2.877896	0
TSEN54	283989	-2.6107361	0
SNORD117	692233	-2.8293664	0
FBRSL1	57666	-2.243737	0
MIR23A	407010	-2.193306	0
SNORA23	677808	-3.2678993	0
PIGW	284098	-2.149463	0
SNORA8	654320	-4.5891285	0.02020202
LOC100288162	100288162	-2.2554677	0.02020202
RFNG	5986	-2.2187188	0.02020202
SNORA9	677798	-2.9003572	0.02020202
RXRB	6257	-2.4855814	0.02020202
CHSY1	22856	-2.0405383	0.030303031
NCOR2	9612	-2.1620538	0.030303031
STX16	8675	-2.0439882	0.030303031
SNX25	83891	-2.3050969	0.030303031
ZNF524	147807	-3.4322982	0.030303031
SNORA55	677834	-2.3716238	0.04040404
SSH1	54434	-2.002753	0.04040404
**Up_Gene Symbol**			
RIIAD1	284485	3.8731875	0
LOC101929741	101929741	7.2783465	0
LINC00240	100133205	3.3969362	0
LINC00845	100507058	5.435211	0
TRIM64B	642446	6.6734176	0
C17orf98	388381	5.1731205	0
PYY	5697	4.068584	0
NAPSA	9476	4.2063127	0
MAGEB10	139422	4.031648	0
ITIH6	347365	6.3592973	0
UGT2B15	7366	10.825695	0.02020202
LY6D	8581	2.8018305	0.02020202
LOC100507377	100507377	3.4407458	0.02020202
LOC100506700	100506700	6.667328	0.030303031

^*^These genes were statistically down- or up-regulated in RAB39A-silenced and slowly growing spheres.

^**^Replicate analysis (Strand NGS); Moderate T test followed by Westfall Young correction was performed between RAB39A-knockdowns and the other spheres. Genes showing significant changes in expression in RAB39A-silenced and slowly growing spheres. RNA-seq analyses of 143B and HeLa cell spheres were used to obtain transcription profiles of slow growing cancer spheres in which expression of RAB39A had been silenced (shRAB(4) and (5)), for comparison to fast growing spheres, including parental cultures, RAB39A-overexpressing cells (RAB39), and transduced controls (V5 and shCont). Genes that were up-regulated or down-regulated > 2-fold were selected as candidates; these were statistically confirmed in the analysis of both 143B and HeLa spheres. T-test followed by Westfall Young correction was performed using “Replicate analysis” (Strand NGS software version 2.0).

**Table 2:** Pathways affected by RAB39A reduction in CSC spheres

Down_Pathway	Pathway Entities ^#^	Matched Entities ^*^	*p*-value ^**^
Hs_Notch_Signaling_Pathway_WP268_70096	46	2	2.88E-04
Hs_Vitamin_D_Metabolism_WP1531_82221	11	1	0.005967482
Hs_Post-translational_modification-_synthesis_of_GPI- anchored_proteins_WP1887_83233	26	1	0.013513307
Hs_Nuclear_Receptors_WP170_71083	38	1	0.020471161
Hs_Signaling_by_Retinoic_Acid_WP3323_83286	42	1	0.0226026
Hs_Vitamin_A_and_Carotenoid_Metabolism_WP716_ 83589	43	1	0.023134766
Hs_miRNAs_involved_in_DNA_damage_response_ WP1545_84697	69	1	0.024729608
Hs_Transcriptional_activity_of_SMAD2-SMAD3-SMAD4_heterotrimer_WP2755_83472	48	1	0.024729608
Hs_Aryl_Hydrocarbon_Receptor_WP2586_85335	48	1	0.025260668
Hs_Pre-NOTCH_Expression_and_Processing_WP2786_83418	68	1	0.027382156
**Up_Pathway**			
Hs_Tamoxifen_metabolism_WP691_85084	21	1	0.007245274
Hs_Glucuronidation_WP698_79224	26	1	0.00896314
Hs_Endothelin_Pathways_WP2197_74852	33	1	0.011020934
Hs_Surfactant_metabolism_WP3579_83469	36	1	0.011363514
Hs_Phase_II_conjugation_WP1880_83364	97	1	0.031381793

^#^The gene number on the pathway, according to the database.

^*^The number of genes that were statistically down- or up-regulated in poorly grown cancer-spheres.

^**^Pathway analysis (Strand NGS); Fisher exact test was performed between the Entity List of down-regulated genes and WikiPathway. Pathways affected by RAB39A reduction in CSC spheres. Using RNA-seq analysis of 143B and HeLa sphere cultures, we profiled transcription in RAB39A-knockdown spheres (shRAB (4) and (5)) in respect to untreated spheres, RAB39A-overexpressing spheres (RAB39), and controls (V5 for RAB39, and shCont for shRAB (4) and (5)). We selected genes with 2-fold higher or lower expression in RAB39A silenced spheres, and that were also confirmed to be statistically reproducible in a HeLa sphere model. Then, the pathways associated with the selected candidate genes were identified by using the “Pathway Analysis” software package (Strand NGS software version 2.0).

**Figure 3:**
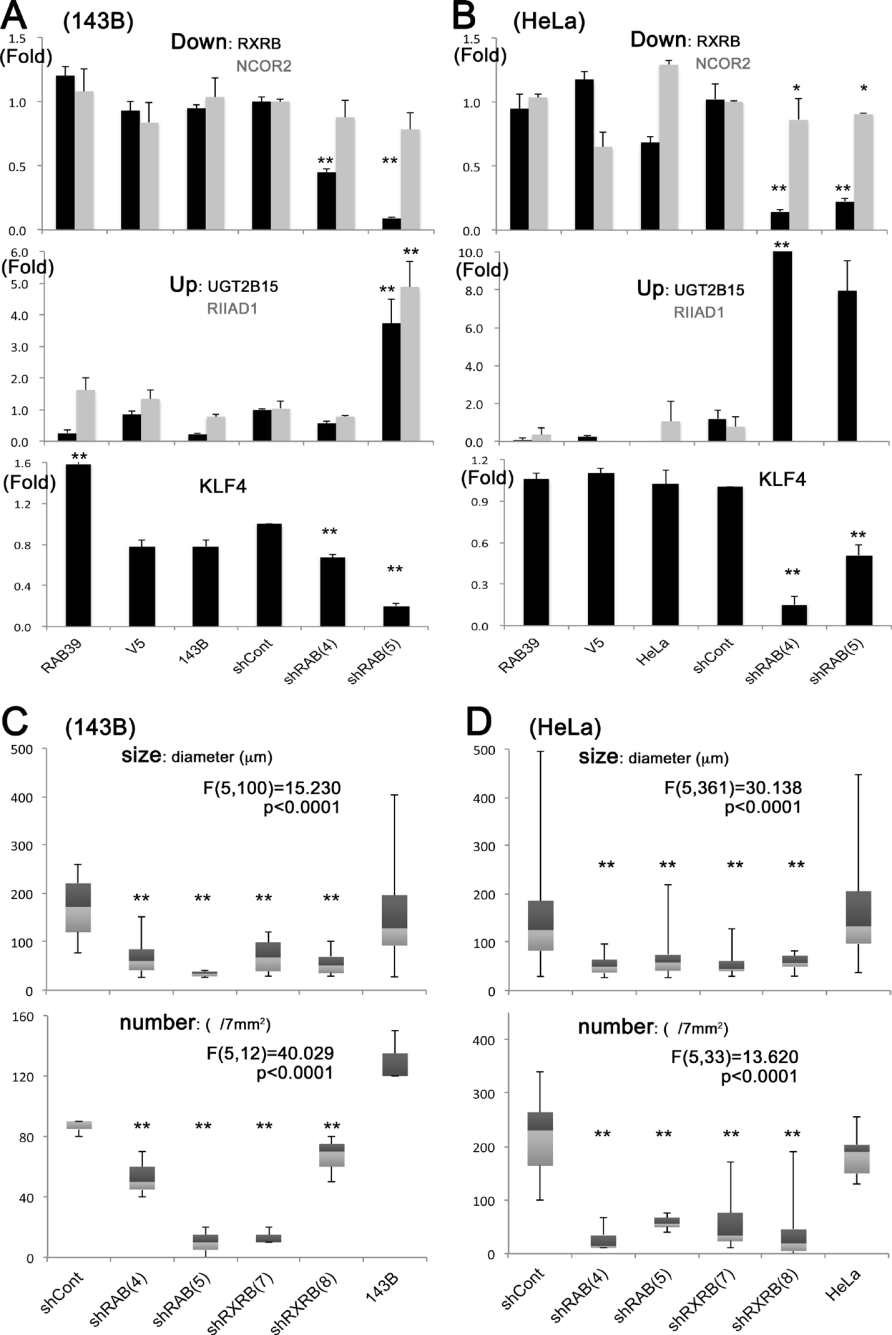
RXRB expression is strongly inhibited in RAB39A-silenced and slowly growing spheres, and its knockdown further impairs sphere formation (**A**) Slowly growing spheres that were formed by 143B cells silenced for *RAB39A* expression with shRAB (4) and (5) (verified by qR-PCR). The upper panel shows *RXRB* and *NCOR2* inhibition of expression (black and gray bars respectively). ANOVA: F (5, 15) = 113.722, *p* < 0.0001 for *RXRB*; F (5, 18) = 0.761, *p* = 0.5893 for *NCOR2*. The middle panel shows *UGT2B15* and *RIIAD1* induction of expression (black and gray bars, respectively). ANOVA: F (5, 18) = 16.529, *p* < 0.0001 for *UGT2B15*; F (5, 12) = 16.074, *p* < 0.0001 for *RIIAD1*. On the lower panel, *KLF4* is enhanced in *RAB39A*-induced cells and inhibited in the silenced cells. ANOVA: F(5,13) = 188.165, *p* < 0.0001 for *KLF4*. (**B**) qRT-PCR validation in HeLa cells was performed similarly to A. ANOVA: F (5, 16) = 36.718, *p* < 0.0001 for *RXRB*; F (5, 18) = 6.449, *p* < 0.0001 for *NCOR2*; F (5, 18) = 10.707, *p* < 0.0001 for *UGT2B15*; F (5, 12) = 0.828, *p* = 0.5535 for *RIIAD1*; F(5,13) = 56.852, *p* < 0.0001 for *KLF4*. (**C**) Silencing of *RXRB* with *shRXRB* (7) and (8) in 143B cells reduces sphere size (diameter) and number to a similar extent as that obtained by *RAB39A* silencing with shRAB (4) and (5). ANOVA: F (5,100) = 15.230, *p* < 0.0001 for size; F (5, 12) = 40.029, *p* < 0.0001 for number. (**D**) Sphere-forming assay in HeLa cells, performed similarly to C. ANOVA: F (5,361) = 30.138, *p* < 0.0001 for size; F (5, 33) = 13.620, *p* < 0.0001 for number. Statistical evaluations in A–D were performed with one-way factorial ANOVA accompanied by Fisher’s PLSD test. In Fisher’s PLSD, ^**^p < 0.05 vs. parental and controls (V5 or shCont), ^*^p < 0.05 vs. parental cells.

*RXRB* inhibition resulting from *RAB39A* silencing was also confirmed by qRT-PCR analysis in spheres obtained from both 143B and HeLa cells (Figure 3A and 3B), and in some cases was associated with the inhibition of *NCOR2*. *UGT2B15* and *RIIAD1* were increased, although not in all the *RAB39A* silenced spheres (Figure 3A and 3B). The stemness-related genes *KLF4*, *SOX2*, *NANOG* and *POU5F1*/*OCT3* were analyzed and only *KLF4* was significantly reduced in *RAB39A* silenced spheres from both 143B and HeLa cells; this was also confirmed by qRT-PCR analysis (Figure 3A and 3B). Sphere-forming ability was affected by the silencing of *RXRB* in both 143B and HeLa cells that were treated with 2 different knockdowns, shRXRB (7) and (8), and similarly, by the silencing of *RAB39A* in cells treated with either shRAB (4) or shRAB (5) (Figure 3C and 3D).

**Figure 4:**
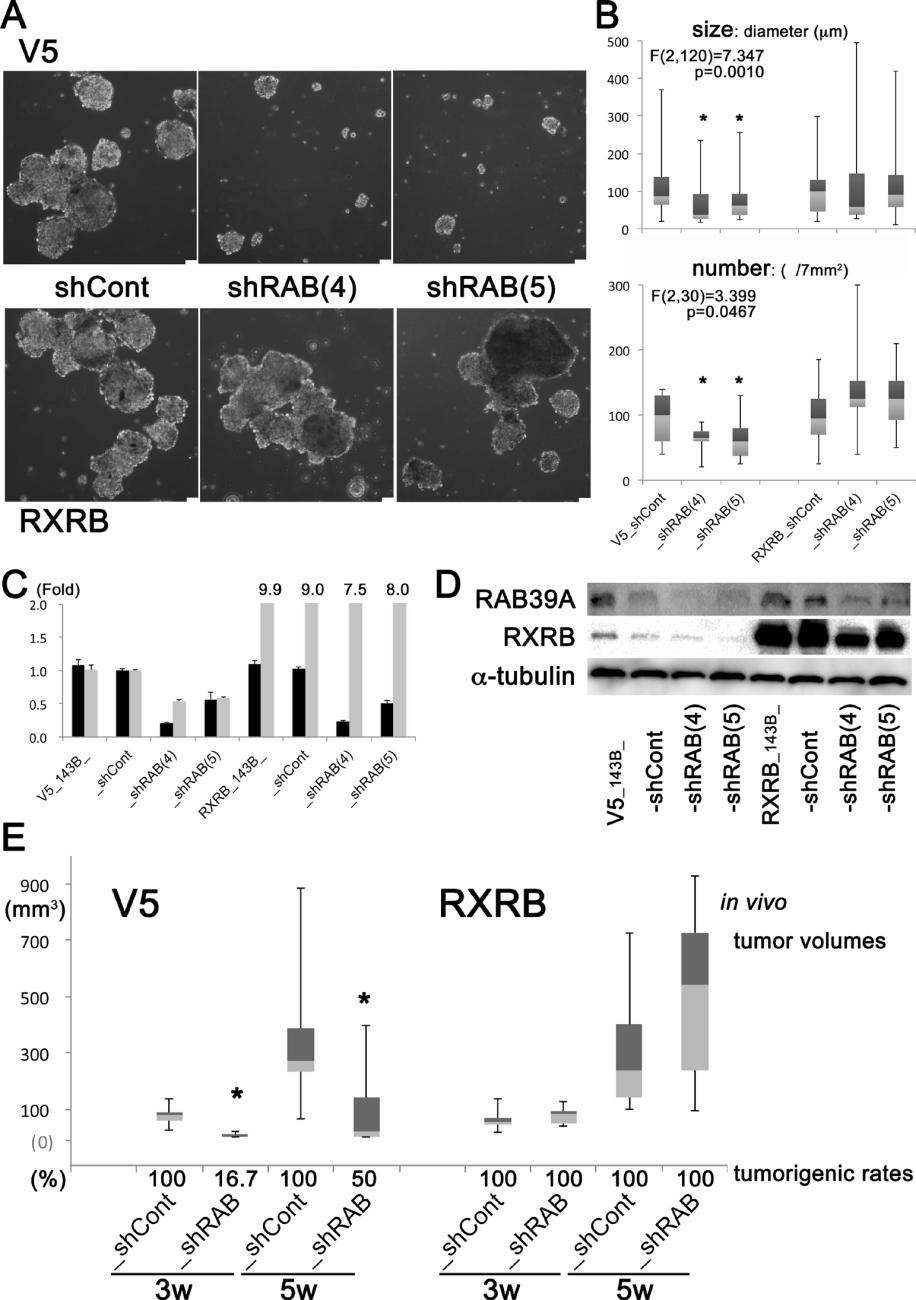
Viral transduction of RAB39A-silenced cells with RXRB restores spherogenicity *in vitro*, and tumorigenicity *in vivo* (**A**) Silencing of *RAB39A* expression using a V5 sequence-containing vector strongly affected sphere formation (shRAB (4) and (5) silenced variants vs. shCont) of 143B cells. This effect was lost when cells were transduced with an *RXRB* containing viral vector. Scale bar, 75 µm. (**B**) Sphere size (diameter) and number of spheres are shown in the panel A. ANOVA: In V5 group, F (2,120) = 7.347, *p* = 0.0010 for size; F (2,30) = 3.399, *p* = 0.0467 for number; ^*^p < 0.05, Fisher’s PLSD vs. shCont. In RXRB group, sphere-forming ability was completely restored with *RAB39A*-deficient cells. F (2,165) = 0.214, *p* = 0.8077 for size; F (2, 30) = 1.070, *p* = 0.3557 for number. (**C**) qRT-PCR analysis of *RAB39A* and *RXRB* (black and gray bars, respectively) in 143B transduced cells for *RAB39A* inhibition and *RXRB* induction. (**D**) Data shown in panel C for *RAB39A* and *RXRB* were confirmed by Western blot analysis. *RXRB* transduced variants have a higher level of the RXRB protein. (**E**) After subcutaneous injection of mice with 10^5^ tumor cells that were previously transduced with V5- or *RXRB*-carrying vectors, tumor volumes and tumorigenic rates were measured. In the V5-control group, tumor volumes were significantly smaller in *RAB39A*-silenced tumors (shRAB) (at 3 weeks, *p* = 0.0033; at 5 weeks, *p* = 0.0360, Mann-Whitney *U*-test vs. shCont). The induced expression of *RXRB* restored the tumorigenicity of *RAB39A*-silenced 143B, as shCont and shRAB showed similar tumor volumes and tumorigenic rates. shRAB and shCont correspond to shRAB (4) and shCont in the *in vitro* experiments.

To confirm that RXRB is a downstream effector of RAB39A that fosters cancer stemness, we induced overexpression of *RXRB* in *RAB39A*-knockdown 143B cells and evaluated their sphere-formation ability and tumorigenesis *in vivo*. Overexpression of *RXRB* in *RAB39A*-deficient cells completely restored the sphere-forming ability (Figure 4A–B). *RXRB* overexpression in the analyzed models was confirmed both by qRT-PCR (Figure 4C) and western blot analysis (Figure 4D). Additionally, overexpression of *RXRB* restored the tumorigenicity of *RAB39A*-knockdown cells in xenografted mice (Figure 4E, Supplementary Figure 3B). In the V5 controls from 143B cells, *RAB39A*-knockdown (V5_shRAB) caused significant reduction in tumor volume and rates of tumorigenesis compared to control cells (V5_shCont). *RXRB* overexpression in the RXRB_shRAB cells restored tumor growth potential to the similar or more level as the RXRB_shCont cells. Our data clearly demonstrated that RAB39A and its downstream molecular effector RXRB fostered cancer stemness and tumorigenesis, and that RAB39A-RXRB axis is a potential target for cancer therapies.

### RAB39A expression is crucial for the pathogenesis of different malignancies

**Figure 5:**
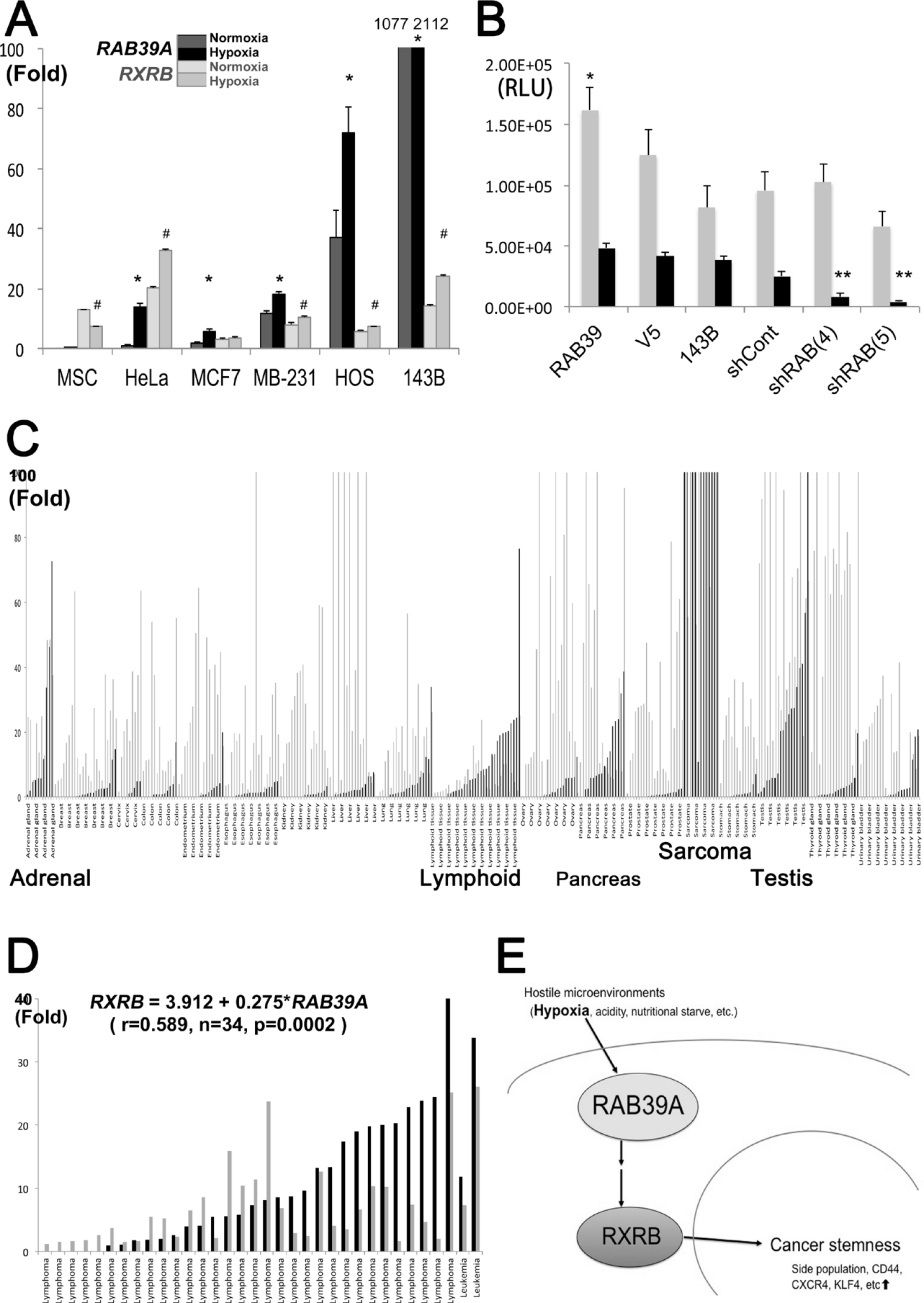
RAB39A function and expression in cancer (**A**) Fold changes in expression of *RAB39A* (2 kinds of black bars) and *RXRB* (2 kinds of gray bars) under normoxia (lighter blacks or grays), and under 1% O_2_ hypoxia (darker blacks or grays), in various types of cancer cell. Hypoxia induces *RAB39A* and *RXRB*, which were confirmed by qRT-PCR; ∆Ct value of HeLa cells under normoxia is shown as 1 or 20, respectively corresponding to *RAB39A* or *RXRB*. ^*^(*RAB39A*) or # (*RXRB*), *p* < 0.05, Mann-Whitney *U*-test vs. each normoxic culture. (**B**) Viability under normoxia and hypoxia (gray and black bars, respectively) of *RAB39A*-silenced spheres obtained from different *RAB39A* variants was measured as luminescence values in 143B cells. The data were analyzed using one-way factorial ANOVA coupled to Fisher’s PLSD test. For normoxia (gray bars), ANOVA: F (5, 30) = 4.050, *p* = 0.0063, ^*^p < 0.05, significant by Fisher’s PLSD vs. 143B cells. For hypoxia (black bars), ANOVA: F (5, 24) = 28.864, *p* < 0.0001, ^**^p < 0.05, vs. shCont and parental 143B cells. (**C**) qRT-PCR analysis of *RAB39A* and *RXRB* expression in various human malignancies. *RAB39A* (black bars) was mainly expressed in sarcomas and malignancies originating from adrenal, lymphoid, pancreas and testicular tissues, whereas *RXRB* (gray bars) was more widely expressed, irrespective of tumor histotypes. Each *RAB39A* or *RXRB* value in the control normal sample was indicated as 1 or 20, respectively, for the representation. Generally, ∆Ct values to ACTB were 5–8 fold higher in *RXRB* than that of *RAB39A* in TaqMan^®^ qRT-PCR. (**D**) *RAB39A* and *RXRB* expressions in lymphoid malignancies. The fold increases of *RAB39A* (black bars) and *RXRB* (gray bars) are shown, and the positive correlation is significantly indicated (*RXRB* = 3.912 + 0.275^*^RAB39A, r = 0.589, *n* = 34, *p* = 0.0002; Pearson’s correlation coefficient). (**E**) Hostile microenvironments like hypoxia induce RAB39A and its downstream effector RXRB, fostering cancer stemness and tumorigenesis.

Hypoxia triggers cancer glycolysis and lysosomal acidification and is often associated with enhanced cancer stemness as it is a determinant of CSC evolution *in vivo* and in CSC niches [16]. As RAB39A is involved in lysosome maturation [8, 9] and, according to our presented data, also in cancer stemness, we investigated whether hypoxia might modulate the expressions of *RAB39A* and *RXRB*, and mediate CSC survival under hypoxic conditions. We found that under hypoxia, *RAB39A* expression significantly increased in various cancer cell types, including sarcoma cells. *RXRB* was also induced by hypoxia in 5 out of 6 cancer cells, especially in HeLa and 143B cells, even though it was reduced in MSC (Figure 5A). Furthermore, the silencing of *RAB39A* significantly altered the viability of CSCs that were cultured under low oxygen tension conditions (Figure 5B), indicating that therapeutic targeting of *RAB39A* might selectively impair CSCs in hypoxic areas of tumors.

To further validate RAB39A and RXRB as potential targets for cancer therapy, we analyzed their expression levels in various malignancies from clinical samples. To date, a high level of cytoplasmic expression of RXRB has been demonstrated in thyroid carcinoma [17]; however, the expression of *RAB39A* has not been explored. Here, we found that *RAB39A* was expressed in malignancies originating from adrenal, lymphoid, pancreas and testicular tissues, with a higher level of expression (often 100-fold higher) in clinical sarcoma samples than other types of cancers. *RXRB* was expressed in different cancers, irrespective of the tumor histotype (Figure 5C). Our data conformed to The Cancer Genome Atlas (TCGA) database, indicating that *RAB39A* was highly expressed in testicular germ cell tumor, glioblastoma, glioma, pheochromocytoma, lymphoma, leukemia, invasive breast cancers and sarcomas, etc; and that *RXRB* was expressed more abundantly in all cancer histotypes (http://www.cbioportal.org/cross_cancer.do?session_id=59d5a712498e5df2e296350a; Supplementary Figure 4A and 4B). Interestingly, *RXRB* expression levels were significantly correlated with *RAB39A* expression levels only in tumor tissues (r = 0.205, *n* = 325, *p* = 0.0002) and not in normal tissues (r = 0.202, *n* = 69, *p* = 0.0968). The highest level of correlation was found in lymphoid malignancies (*RXRB* = 3.912 + 0.275*RAB39A, r = 0.589, *n* = 34, *p* = 0.0002; Figure 5D). Together, the clinical sample data strengthen our hypothesis that RXRB is a downstream effector of RAB39A, and that RAB39A-targeting treatments will selectively affect cancer cells and be unlikely to cause undue side effects in normal tissues.

## DISCUSSION

In the present study, we used global transcriptomics, PCA and shRNA-based function screening to investigate the roles of *RAB39A*, *CPVL*, *NUP210, LHX2, KCNG3*, *SLITRK5*, *FXYD6* and *PRAME* expression in cancer cells, including CSCs, under both acidic and neutral microenvironmental conditions. Our results indicate that these genes are selective therapeutic targets in cancer cells. Some of the candidate genes have previously been shown to be involved in cancer development [18–27], confirming the reliability of the procedure used here to detect cancer-specific targets. Among the candidate genes, *RAB39A* appears to be very promising since its knockdown had a clear impact on tumorigenicity *in vivo*, especially during the early steps of tumor formation, when cancer stemness has a major role. Therefore, we suggest that RAB39A is involved in cancer stemness regulation.

Rab proteins are the largest family among the Ras-like small GTPase superfamily, and control vesicular trafficking. Indeed, RAB39A function is associated with late endosomes and lysosomes, and regulates endocytosis and acidification of maturing phagosomes [8, 28]. RAB39A may thus be crucial for tumorigenesis since these processes are also linked to autophagy, which has an established role in cancer [29]. In autophagy, RAB39A negatively regulates LPS-induced autophagosome formation, and secretion of pro-inflammatory compounds, possibly through the involvement of the PI3K pathway [8, 9]. Our previous report showed a clear connection between lysosomal acidification and cancer stemness [15], and we thus speculated that CSCs are particularly good targets for anti-RAB39A therapeutic strategies. In agreement with our speculation, our data clearly demonstrated that targeting *RAB39A* inhibits cancer stemness and tumorigenesis. This is the first time that RAB39A has been studied in cancer biology, with the exception of a report on mouse Neuro2A cells and their involvement in neuronal differentiation, such as retinoic acid-induced neurite morphology [30].

In order to further explore the RAB39A downstream pathways that affect cancer stemness, we used RNA-seq to screen spheres produced by cells with a knockdown of *RAB39A* expression. In combination with a cellular functional analysis following RAB39A knockdown and RXRB augmentation, this strategy enabled us to identify RXRB as a downstream effector of RAB39A that fosters cancer stemness. RXRB is a member of the RXR family of nuclear receptors that mediates the effects of retinoic acid, and it forms heterodimers with the retinoic acid receptor (RAR), thyroid hormone and VDR, increasing both DNA binding and transcriptional function on their respective response elements [10]. RXRB is widely expressed and plays a crucial role in spermatogenesis [31–33]. Furthermore, the mutual heterodimers between RXR and VDR are involved in cancer developments, and activated Ras-Raf-MAPK-ERK can engage in the classical VDR pathway to modulate variously gene expressions [11]. Ras-like small GTPase activity contained in RAB39A may contribute to cancer developments through RXR and VDR signaling. RXRB is also expressed in various malignancies [17, 34–38] and promotes cell survival/proliferation in triple-negative breast cancer [39], thus indirectly confirming a crucial role of the RAB39A-RXRB axis in modulating cancer stemness. In addition, TCGA database showed that genetic amplification of *RAB39A-RXRB* was often seen in various types of malignancies especially like breast and neuroendocrine prostate cancers (Supplementary Figure 4C), suggesting that the RAB39A-RXRB axis plays a prominent role for development of various malignancies.

Intratumoral hypoxia is a widely accepted characteristic of CSC evolution *in vivo* and in CSC niches [16]. We focused on evaluating the expression of *RAB39A* under hypoxic conditions and the effects of its inhibition. Our data showed that *RAB39A* expression increased in hypoxic cancer cells and that its inhibition significantly reduced the viability of hypoxic CSCs. Hypoxia is a trigger of cancer glycolysis; lysosomal compartmentalization is the most effective detoxification mechanism for high proton intracellular contents associated with glycolysis, and RAB39A activity might therefore be required for lysosomal acidification [8, 9, 28]. Taken together, our data from the preclinical model strongly suggested that targeting the RAB39A-RXRB axis might result in selective impacts on CSCs in hypoxic areas of tumors.

Through analyses of both clinical samples and TCGA database, we confirmed that *RAB39A* was expressed in sarcomas and malignancies of lymphoid, adrenal and testicular tissues; and *RXRB* abundance was constantly widespread in various cancers, suggesting that RXRB functions with the saturated level in cancers. Induced *RXRB* showed the restoration of both spherogenesis and tumorigenesis, but further increase of its expression couldn’t significantly enhance these abilities. On the contrary, silencing of *RXRB* or *RAB39A* inhibited the spherogenicity with a major and significant effect, possibly through impairment of the stemness. These data suggest that RXRB functioning protein is at almost saturated activity and that RXRB-mediated cancer stemness has been already maximized in various cancers. It is why inhibiting RAB39A or RXRB can critically affect cancer stemness. In the clinical sample analysis, *RXRB* levels were not correlated with *RAB39A* expression levels in normal tissues, but a significant correlation was present in tumor tissues. These observations additionally support our hypothesis that RXRB is a downstream effector of RAB39A, and that targeting RAB39A will selectively affect cancer and CSCs and be unlikely to cause undue side effects in normal tissues.

In conclusion, this study has identified and validated RAB39A as a promising therapeutic target in various human malignancies. We demonstrated that RAB39A-RXRB axis plays a prominent role in cancer development and stemness, and that targeting RAB39A and inhibiting its downstream molecular effecter RXRB strongly impairs tumorigenesis and cancer stemness (Figure 5E), with particular regard to musculoskeletal sarcomas and lymphoid malignancies. RAB39A and RXRB thus deserve further investigation that will allow the development of novel drugs for more effective and novel anti-cancer therapeutic strategies.

## MATERIALS AND METHODS

### Cell lines

Human osteosarcoma cells (MG-63, HOS) and normal fibroblasts (TIG-108, TIG-121) were obtained from the Japanese Collection of Research Bioresources (Osaka, Japan). Primary cultures of mesenchymal stromal cells (MSCs) (#305526, #351482, #326162, #367500) were acquired from Lonza (MD). Saos-2 human osteosarcoma cells were obtained from RIKEN BioResource Center (RIKEN BRC, Tsukuba, Japan). HeLa human endocervical carcinoma and 143B human osteosarcoma cells were obtained from the American Type Culture Collection (ATCC, Manassas, VA). Cells were cultured in Dulbecco’s modified Eagle’s medium containing 10% fetal bovine serum, supplemented with penicillin (50 units/mL) and streptomycin (50 mg/mL) with 10 mM 4-(2-hydroxyethyl)-1-piperazineethanesulfonic acid to achieve pH 7.4, or with 10 mM piperazine-1,4-bis(2-ethanesulfonic acid) to achieve pH 6.5. Cells were incubated at 37°C in a humidified chamber supplemented with 5% CO_2_.

### Sphere-forming culture

Briefly, osteosarcoma cells with or without lentiviral transfection were cultured in 6-well plates (30,000 cells/well) coated with polyhema (Sigma-Aldrich) in anchorage-independent conditions. DMEM-F12 complete medium was used at either pH 6.5 or 7.4. Progesterone (20 nM), putrescine (10 mg/mL), sodium selenite (30 nM), apo-transferrin (100 mg/mL), and insulin (25 mg/mL) (Sigma-Aldrich) were added to the complete medium. Fresh human epidermal growth factor (20 ng/mL) and basic fibroblast growth factor (10 ng/mL) (Gibco-Life Technologies) were added twice a week. After 10 days, bright field images were acquired using a Leica DMI4000B (Leica Microsystems). Only spheres with a diameter of more than 20 μm were counted. Analysis of cell diameter was performed with Leica Application Suite Software (Leica Microsystems).

### RNA extraction and RNA-Seq analysis

Total RNA from cell cultures or from spheres was extracted using guanidinium thiocyanate-phenol-chloroform. The total RNA was quantified with a Bioanalyzer (Agilent, Santa Clara, CA) following the manufacturer‘s instructions. A RIN (RNA Integrity Number) of 10 and an A260/A280 ratio over 1.8 were obtained for the total RNA.

Following the manufacturer‘s protocol, the library of template molecules for high throughput DNA sequencing was converted from the total RNA using TruSeq RNA Sample Prep Kit v2 (Illumina, San Diego, CA). For detecting RAB39A-cancer stemness pathways, the library was prepared from the total RNA isolated from spheres, using the Ribo-Zero rRNA Removal Kit (Illumina). The library was quantified with a Bioanalyzer (Agilent) following the manufacturer‘s instructions. The library (4 pM) was subjected to cluster amplification on a Single Read Flow Cell v4 with a cluster generation instrument (Illumina). Sequencing was performed on Genome Analyzer GAIIx for 76 cycles or HiSeq 2500 for 101 cycles using Cycle Sequencing v4 reagents (Illumina).

Image analysis and base calling were performed using Off-Line Basecaller Software 1.6 (Illumina). Human genome build 19 (hg19) was downloaded from the University of California, Santa Cruz genome browser (http://genome.ucsc.edu/) as the analytical reference. Reads were aligned using Strand NGS software (version 2.0, Strand Scientific Intelligence Inc., San Francisco, CA) with the sequence data sets. Transcript coverage for every gene locus was calculated from the total number of filter reads that mapped to exons. These analyses and filtering were performed using default parameters. All the advanced analyses for quantification with TMM normalization algorithm, expression analysis, pathway analysis with WikiPathway data, and principal component analysis were also performed using Strand NGS software. Genes with significantly different expressions were identified by the fold change method (fold change > 2), statistically analyzed by Westfall-Young permutation procedure (*p* < 0.05), and categorized into particular pathways using Pathway analysis (*p* < 0.05). All new data has been deposited in DDBJ/EMBL/GenBank under DRA004087, DRA004091 and DRA005595.

### Plasmid DNA and gene transfer

Lentiviral shRNA vectors for *RAB39A* (shRAB(4) and (5)), RXRB (shRXRB(7) and (8)), non-silencing control (shCont) and the other targets (CPVL, NUP210, LHX2s, KCNG3, SLITRK5, FXYD6 and PRAME) were purchased from Dharmacon (GE Healthcare, UK). Lentiviral cDNA vectors for RAB39A and RXRB were obtained from Dharmacon and OriGene (MD), respectively. V5 sequence-containing pLenti6 vector (Invitrogen, Thermo, MA) without any specific cDNA, was used as the overexpression control. Lentivirus transferring shRNA or cDNA were prepared with Lenti-X™ HTX packaging system (Clontech, Takara Bio, Shiga, Japan) according to the manufacturer’s instructions. HOS, HeLa and 143B cells transferred by >20 MOI of each lentivirus were selected in the presence of 3 μg/ml puromycin or 10 μg/ml blasticidin, expanded and used in the experiments.

### WST-8 assay for cell viability

HOS and HeLa cells were cultured in 96-well plates at a density of 1×10^3^ per well, and cell viability assays were carried out by a Cell Counting Kit-8 (Dojindo Molecular Technologies Inc, Kumamoto, Japan). WST-8 reagent solution was added to each well, and the absorbance at 450 nm (OD450) was measured by a microplate reader after the microplate was incubated with the reagent for 2 hours at 37°C, in accordance with the manufacturer's instructions.

### Mice and xenografting of human osteosarcoma cells

Seven-week-old BALB/c nu/nu nude mice (CLEA Japan, Inc., Tokyo, Japan) transplanted with either 143B human osteosarcoma cells or genetically manipulated variants were used in the experiments. A minimum of 6 mice was xenotransplanted with 143B variant cells in each group. A subcutaneous injection of 1 × 10^6^ cells of 143B or a variant was made into the right dorsal flank of each mouse. After the establishment of palpable tumors (about ≥ 5 days), mouse body weight and external tumor volume were determined twice a week. Tumor volume was calculated using the formula *A*^2^ × *B*/2, where *A* and *B* represents the smallest diameter and the largest diameter, respectively. The use of the animals in the experimental protocols was reviewed and approved by the Committee of Research Center for Animal Life Science in Shiga University of Medical Science (Approved No. 2015-3-8).

In order to evaluate the xenotransplantability of *RAB39A*-knockdown cells, *RAB39A* knockdown and the control 143B cells (shRAB and shCont) were transplanted with serial cell dilutions of 10^6^–10^4^ tumor cells into each left and right backs of 6 mice each, respectively (a total of 18 mice was used). After xenotransplanting each dilution of cells, tumor volumes and tumorigenic rates were measured in shRAB and shCont 143B tumors at 3 and 5 weeks. shRAB corresponds to shRAB(4) in the *in vitro* experiments.

After transduction with V5 or RXRB carrying vectors (labeled as V5_ or RXRB_) in 143B cells, tumorigenicity for *RAB39A* knockdown and the control cells (labeled as _shRAB or _shCont) were evaluated. In this case, referring to the previous data using serial cell dilution transplants, 10^5^ tumor cells of 2 pairs of the knockdown variants (V5_shRAB & _shCont; and RXRB_shRAB &_shCont) were xenotransplanted into the backs of 6 mice (a total of 12 mice was used), and tumor volumes and tumorigenic rates were measured at 3 and 5 weeks.

### Quantitative reverse transcription polymerase chain reaction (qRT-PCR)

Using the acid guanidinium thiocyanate-phenol-chloroform method, total RNAs were obtained from cultured cells and 13 clinical sarcoma samples, which had been collected and banked with the written informed consent of patients and after approval by the Ethics Committee of Istituto Ortopedico Rizzoli (No. 0033626), and cDNA were produced by SuperScript^®^ VILO™ Master Mix (Thermo). TissueScan^™^ Cancer and Normal Tissue cDNA Arrays (#CSRT103, OriGene, MD) were applied for evaluating ∼400 clinically human cancer tissues, covering different malignancies of adrenal grand, breast, cervix, colon, endometrium, esophagus, kidney, liver, lung, lymphoid tissue, ovary, pancreas, prostate, stomach, testis, thyroid grand, urinary bladder and uterus (described precisely in http://www.origene.com/qPCR/Tissue-qPCR-Arrays.aspx). Quantitative PCR was performed using a TaqMan^®^ Fast Advanced master mix and StepOnePlus^™^ Real-Time PCR system (Thermo). Gene expression was normalized with human beta actin (*ACTB*: #4326315E) as an endogenous control in each tube, and the relative levels were calculated using the ΔΔCt model [40]. Probe and primer sets were prepared for human *RAB39A* (Hs00380029_m1), *RXRB* (Hs00232774_m1), *CPVL* (Hs01073862_m1), *NUP210* (Hs00227779_m1), *LHX2* (Hs00180351_m1), *NCOR2* (Hs00196955_m1), *UGT2B15* (Hs00870076_s1), *RIIAD1* (Hs01380113_g1) and *KLF4* (Hs00358836_m1); and purchased from Thermo. Each assay was repeated at least three times.

### Side population and stem-related surface maker analyses

To detect cancer stem-like cells, we carried out analyses for the side population (SP) and stem-related cell surface markers, such as CD133, CD44 v5 and CXCR4. Vybrant^®^ DyeCycle™ Violet staining (10 μM) of human stem cells enabled SP fractions to be measured by contrast with those pre-incubated with 10 μM fumitremorgin C that inhibits ABCG2 drug transportation. The cells were washed with PBS, harvested by trypsin, and reconstituted to a final concentration of 1 × 10^6^ cells/ml in Live cell imaging^®^ reagents (Life Technologies, Thermo), together with the DyeCycle™ staining reagent for 30 min, Then, the cells were analyzed under 405 nm excitation in an Attune^®^ Acoustic Focusing Cytometer (Thermo).

For stem-related cell surface marker analysis, the antibody for CD133 (AC133, Miltenyi Biotec, CA) or CD44 v9 (RV3, Cosmo Bio, Tokyo, Japan) was detected using Alexa Fluor^®^ 488 goat anti-mouse or anti-rat IgG (H+L), or CXCR4 (CD184, Miltenyi) pre-conjugated with phycoerythrin. Anti-human CD44 v9 (RV3) specifically detects CD44 variant isoforms (CD44 v8–10), which contribute to the defense against reactive oxygen species by promoting cystine uptake for reduced glutathione, and which drives tumor growth, chemoresistance and metastasis [41, 42]. From a preliminary analysis, CD44 v9 and CXCR4 were known to be abundant in 143B and HeLa cells, respectively, and were therefore used to evaluate each stem-like fraction; CD133 staining was too subtle to detect or to evaluate intensities in either cell type (Supplementary Figure 2A). Harvested cells (1 x 10^6^) were stained, resuspended in 1 ml Live cell imaging^®^ reagents (Thermo), and gated in a forward scatter vs. side scatter, excluding debris or cell aggregations. Then, the gated cell populations were evaluated for expression of each surface maker expression under 488 nm excitation using an Attune^®^ Acoustic Focusing Cytometer (Thermo).

### Immunoblotting

Cells were lysed in Laemmli-SDS buffer; the solution was subjected to SDS-polyacrylamide gel electrophoresis, and then electro-transferred to membrane filters (Immuno-Blot PVDF membranes, Bio-Rad Laboratories, Richmond, CA). The filters were incubated overnight with a primary antibody of RAB39A (#13355-1-AP, Proteintech, IL), RXRB (#8715, Cell Signaling Technology, MA) or α-tubulin (#T9026, DM1A, Sigma-Aldrich, MO) in TBS-T containing 2% bovine serum albumin and incubated for 1 hour in horseradish peroxidase-conjugated anti-rabbit or anti-mouse secondary antibodies (Cell Signaling Technology) diluted 1:10,000 in TBS-T containing 2% bovine serum albumin. Immunoreactivity was detected using the Luminata Classico Western HRP substrate (Millipore Corporation) and an LAS4000 bioimager (Fujifilm, Tokyo, Japan).

### Evaluation for sphere viability

Induced and reduced RAB39A variants, RAB39, V5, parental, shCont, shRAB(4) and (5), of 143B and HeLa cells were seeded at 3000 cells/well into non-adhesively coated 96 well plates (Low Cell Attachment Plate PrimeSurface^®^ MS-9096V, Sumitomo Bakelite, Tokyo, Japan), and incubated in complete DMEM-F12 medium for sphere-forming culture, under normoxic and hypoxic (1% O_2_) conditions. The survival of spheres was analyzed on Day 3 (72 hours after seeding) using a CellTiter-Glo^®^ 3D Cell Viability Assay, as a luminescence value (Promega, Madison, WI).

### Statistical analysis

RNA-seq data analysis was performed using Strand NGS software version 2.0 (Strand Scientific Intelligence Inc.). Other statistical analyses were performed with StatView™ version 5.0 software for Windows (SAS Institute Inc). Results are reported as means ± standard error. One-way factorial ANOVA accompanied by Fisher’s PLSD test was used to compare multiple group means. Non-parametric Mann-Whitney *U* tests were used to compare two group means. Pearson’s correlation coefficient tests were used to identify significant correlations between *RAB39A* and *RXRB* expression in tumor and normal samples. A *p*-value of < 0.05 was considered statistically significant.

## SUPPLEMENTARY MATERIALS FIGURES AND TABLES





## Abbreviations

(RAB39A): Ras-Related Protein RAB39A
(RXRB): Retinoid X Receptor Beta
(CSC): cancer stem cell
(PCA): principal component analysis
(Fb): fibroblast
(MSC): mesenchymal stem cell
(SP): side population
(qRT-PCR): quantitative reverse transcription polymerase chain reaction
(VDR): vitamin D receptor
(RAR): retinoic acid receptor
