# Supplementary material for: Prominent role of RAB39A-RXRB axis in cancer development and stemness
Source: Oncotarget. 2018 Jan 4;9(11):9852–66. doi: 10.18632/oncotarget.23955 (PMC5839406; doi:10.18632/oncotarget.23955)
Supplement: Supplementary file 1 [file oncotarget-09-9852-s001.pdf]

Prominent role of RAB39A-RXRB axis in cancer development and stemness

SUPPLEMENTARY MATERIALS

A

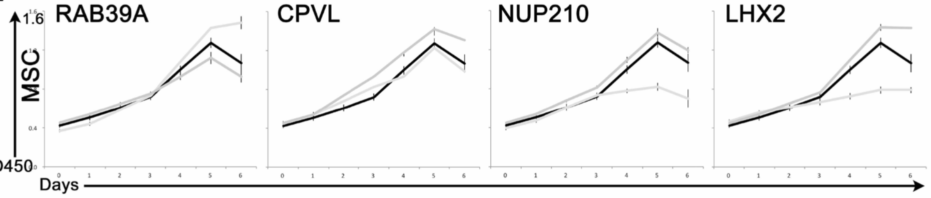

B

Gene Symbol	Description	Gene ID	Component 1 (17.68%)
**KCNG3**	Potassium Voltage-Gated Channel Modifier Subfamily G Member 3	ENSG00000171126	0.016226687
**SLITRK5**	SLIT And NTRK Like Family Member 5	ENSG00000165300	0.01600506
**FXYD6**	FXYD Domain Containing Ion Transport Regulator 6	ENSG00000137726	0.015698582
**PRAME**	Preferentially Expressed Antigen In Melanoma	ENSG00000185686	0.015601972

C

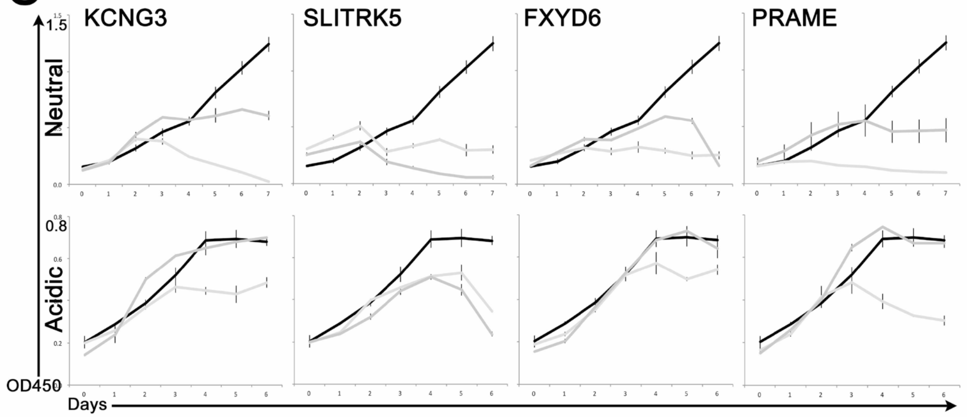

Supplementary Figure 1: Targeting effects in mesenchymal stem cells and four additional targeting candidates. (**A**) Knock-down of prioritized four candidate genes in MSCs did not significantly affect cell growth under neutral pH conditions, especially for *RAB39A* and *CPVL*; (**B**) *KCNG3, SLITRK5, FXYD6,* and *PRAME* were identified as second-choice candidate genes for the targeting of cancer cells and CSCs; (**C**) Knock-down of *KCNG3, SLITRK5, FXYD6* and *PRAME* inhibited cell growth in neutral (pH 7.4) conditions but not under acidic conditions (pH 6.8) in HOS human osteosarcoma cells.

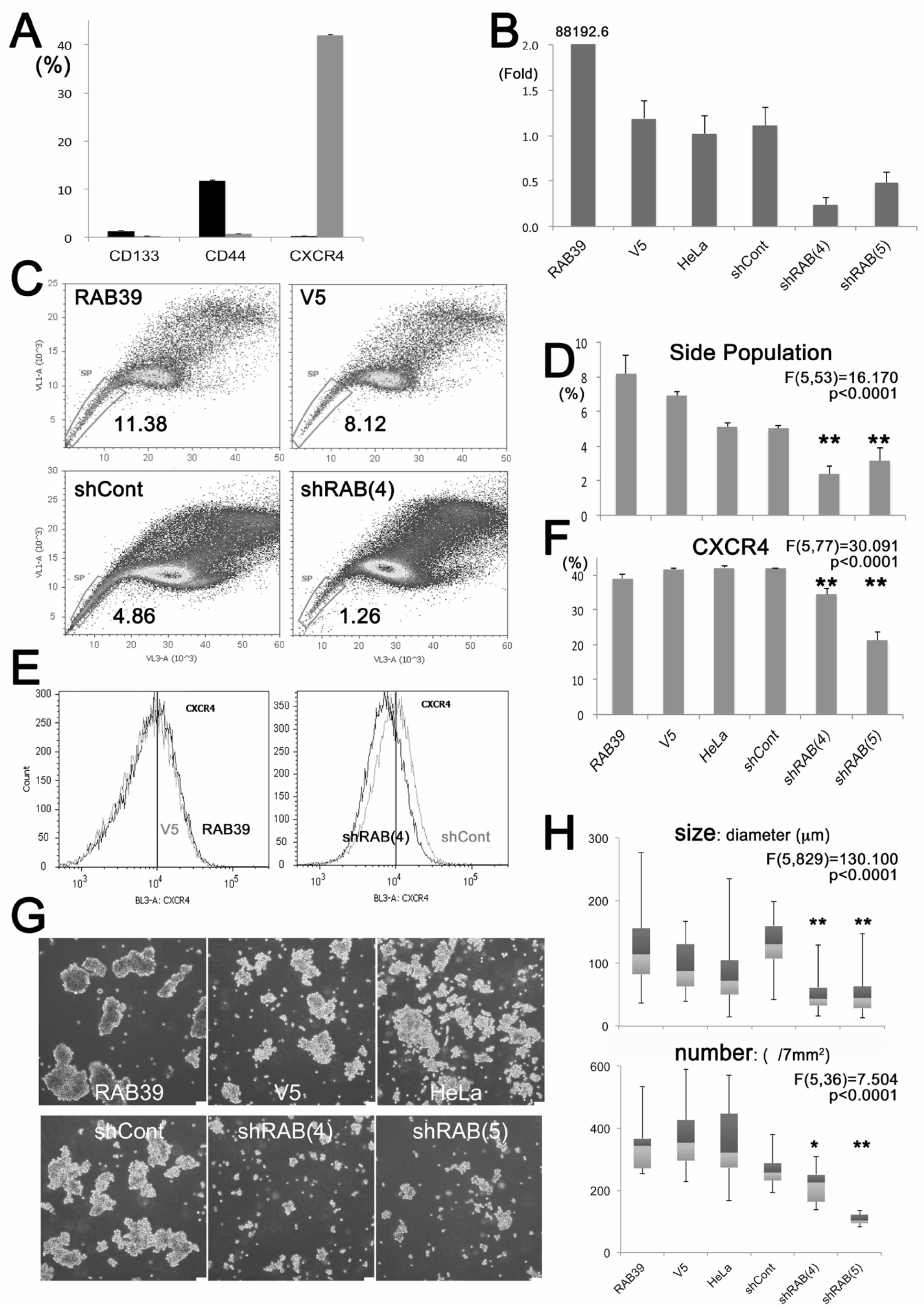

Supplementary Figure 2: Knockdown of *RAB39A* reduces stemness of HeLa cells. (**A**) CD133, CD44 v9 and CXCR4 expression was evaluated by flow cytometry in 143B (black bars) and HeLa (gray bars) cells. The analyses indicated CD44 v9 and CXCR4 were stemness markers for 143B and HeLa, respectively; (**B**) qRT-PCR analysis of *RAB39A* expression using shRAB(4) and (5) knockdown of *RAB39A*. *RAB39A* expression increased by 80,000-fold in cells with over-expression variant (RAB39) compared to the controls (V5 and shCont) and parental cells. (**C**) Flow cytometry analysis showed SP cells increased in number after induced *RAB39A* overexpression, and fell when *RAB39A* expression was knocked down. RAB39 or V5 correspond to cells transfected with *RAB39A* or V5 control. Knockdown of *RAB39A* and the control are shown as shRAB(4) and shCont, respectively. (**D**) Graphic representation of data shown in panel (C) In *RAB39A* silenced cells, the SP was significantly reduced. (**E**) Flow cytometric analysis of CXCR4 expression in RAB39 (black line) and V5 (gray line) cells (left panel), and in shRAB(4) (black line) and shCont (gray line) cells (right panel). (**F**) Graphic representation of the data shown in panel E. In *RAB39A* knockdown cells, CXCR4-positive populations were significantly reduced. (**G**) Sphere formation was reduced after knockdown of *RAB39A* in shRAB(4) and (5) cells. Scale bar, 75 μm. (**H**) After knockdown of *RAB39A*, sphere size (diameter) and number were significantly reduced. $^{**}p < 0.05$, significant by Fisher's PLSD, vs. shCont and parental HeLa cells. $^{*}p < 0.05$, vs. parental HeLa cells.

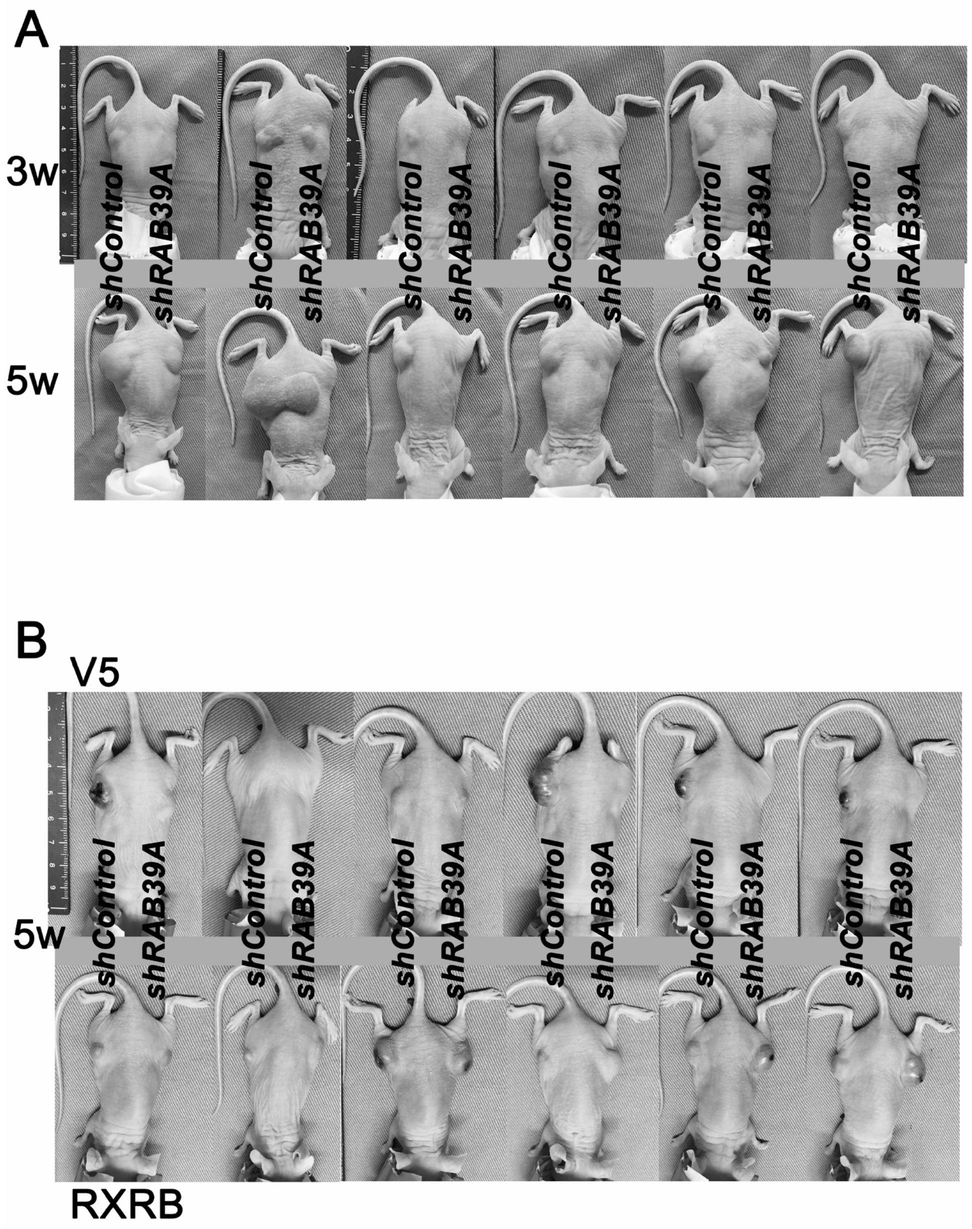

Supplementary Figure 3: *RAB39A* knockdown impairs tumorigenicity but is rescued by *RXRB* overexpression. (**A**) *RAB39A* knockdown 143B cells (shRAB39A, corresponding to shRAB(4) in the in vitro experiments) were subcutaneously injected into the left flank, and control cells were injected into the right flank (*shControl*, corresponding to shCont in in vitro experiments). Photographs of all 6 mice at 3 and 5 weeks after injection of 10^5 tumor cells are shown. (**B**) In the V5 control (upper line of images), the *RAB39A* knockdown 143B cells (shRAB39A) showed a lower rate of tumorigenicity than control cells (*shControl*). RXRB overexpression (lower lined photos) caused similar levels of tumorigenicity in both types of knockdown cell. Photographs of all 6 mice at 5 weeks after injection of 10^5 tumor cells are shown.

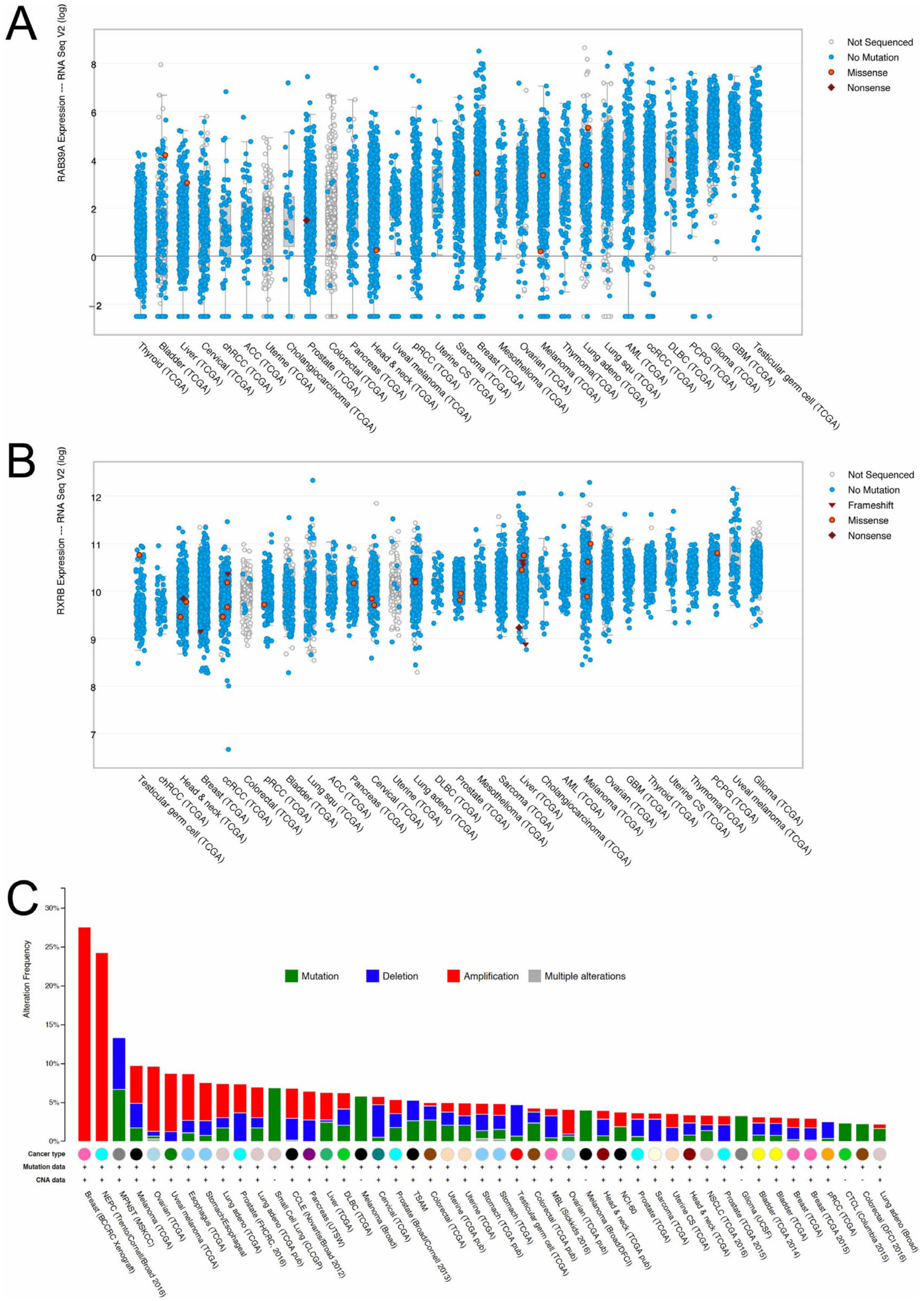

Supplementary Figure 4: RAB39A-RXRB axis in TCGA database: (**A**) RAB39A expression; (**B**) RXRB expression; and (**C**) Mutation spectrum of RAB39A-RXRB axis in the database of The Cancer Genome Atlas (TCGA).

Supplementary Table 1: The 10 most up-regulated pathways in sarcoma cells and CSCs

Pathway	Pathway Entities[#]	Matched Entities[*]	*p*-value[**]
Hs_G1_to_S_cell_cycle_control_WP45_71377	68	17	0
Hs_Mitotic_Prometaphase_WP2652_76819	98	35	0
Hs_RB_in_Cancer_WP2446_76353	87	32	0
Hs_DNA_Replication_WP466_76196	42	14	0
Hs_Synthesis_of_DNA_WP1925_76968	94	17	0
Hs_Cell_Cycle_Checkpoints_WP1775_76816	115	19	0
Hs_Cell_Cycle_WP179_70629	103	27	0
Hs_S_Phase_WP2772_77049	116	20	1.59E–11
Hs_Mitotic_Metaphase_and_Anaphase_ WP2757_77009	153	30	5.68E–11
Hs_Mitotic_G1-G1-S_phases_WP1858_76928	120	25	6.44E–11

[#]The gene number on the pathway, according to the database.
[*]The number of genes that were statistically up-regulated in sarcoma and CSC under the acidic environment.
[**]Pathway analysis (Strand NGS); Fisher exact test was performed between the Entity List of up-regulated genes and WikiPathway. The enhanced pathways in sarcoma cells and CSCs, under acidic conditions, are mostly related to the cell cycle and to DNA replication and synthesis.

Supplementary Table 2: The 10 most up-regulated pathways in normal cells

Pathway	Pathway Entities#	Matched Entities*	*p*-value**
Hs_TCR_Signaling_Pathway_WP69_72111	92	7	2.88E-04
Hs_TSLP_Signaling_Pathway_WP2203_72104	48	5	4.65E-04
Hs_IL-4_Signaling_Pathway_WP395_74009	55	5	8.23E-04
Hs_Interleukin-1_processing_WP1838_77045	4	2	0.001173588
Hs_Corticotropin-releasing_hormone_WP2355_71393	90	6	0.001649494
Hs_Interleukin-1_signaling_WP1839_76943	38	4	0.001790027
Hs_Sphingolipid_Metabolism_WP1422_71368	21	3	0.003081359
Hs_Toll-like_receptor_signaling_pathway_WP75_72133	102	6	0.00312705
Hs_Regulation_of_toll-like_receptor_signaling_pathway_WP1449_72114	150	6	0.00312705
Hs_Regulation_of_toll-like_receptor_signaling_pathway_WP1449_77378	150	6	0.00312705

#The gene number on the pathway, according to the database.
*The number of genes that were statistically up-regulated in fibroblasts and mesenchymal stem cells under the acidic environment.
**Pathway analysis (Strand NGS); Fisher exact test was performed between the Entity List of up-regulated genes and WikiPathway.
The enhanced pathways in normal cells, under acidic conditions, are mostly related to inflammatory pathways.
For the analyses of the data in Supplementary Tables 1-2, we used RNA-seq data from malignant cells, MG-63, HOS, Saos-2, the respective spheres, and from normal cells, two fibroblast cell lines (TIG-108, -121) and four samples of mesenchymal stem cells (Lonza #305526, #351482, #326162, #367500). The levels of transcription under acidic (pH 6.5) conditions were compared between malignant and normal cells. Genes reproducibly more than 2-fold up-regulated were selected into the Entity List. Using the "Pathway Analysis" software package (Strand NGS software version 2.0) from the list, the pathways showing greatest up-regulation were identified in malignant or normal cells, demonstrated in Supplementary Tables 1 and 2, respectively.

Supplementary Table 3: Top 100 genes on component 1 axis of principal component analysis. See_ Supplementary_Table 3
