# Supplementary material for: Prominent role of RAB39A-RXRB axis in cancer development and stemness
Source: Oncotarget. 2018 Jan 4;9(11):9852–66. doi: 10.18632/oncotarget.23955 (PMC5839406; doi:10.18632/oncotarget.23955)
Supplement: Supplementary file 2 [file oncotarget-09-9852-s002.docx]

**Supplementary Table 3: Top 100 genes on component 1 axis of principal component analysis.**

| **Gene Symbol** | **Description** | **Gene ID** | **Component 1 (17.68%)** |
| --- | --- | --- | --- |
| **RAB39** | **RAB39A, Member RAS Oncogene Family** | **ENSG00000179331** | **0.016616153** |
| IGSF3 |  | ENSG00000143061 | 0.016561998 |
| **ARAP2** | *ArfGAP With RhoGAP Domain, Ankyrin Repeat And PH Domain 2* | ENSG00000047365 | 0.016419124 |
| **CPVL** | **Carboxypeptidase, Vitellogenic Like** | **ENSG00000106066** | **0.016415408** |
| **NUP210** | **Nucleoporin 210kDa** | **ENSG00000132182** | **0.016395373** |
| PPARGC1B |  | ENSG00000155846 | 0.016291684 |
| **KCNG3** | **Potassium Voltage-Gated Channel Modifier Subfamily G Member 3** | **ENSG00000171126** | **0.016226687** |
| **LHX2** | **LIM Homeobox 2** | **ENSG00000106689** | **0.01619214** |
| GNRHR2 |  | ENSG00000211451 | 0.016170906 |
| COCH |  | ENSG00000100473 | 0.016164437 |
| KLHL23 |  | ENSG00000213160 | 0.016089475 |
| TBX20 |  | ENSG00000164532 | 0.016079923 |
| PM20D2 |  | ENSG00000146281 | 0.016040817 |
| CDCA4 |  | ENSG00000170779 | 0.016027521 |
| **SLITRK5** | **SLIT And NTRK Like Family Member 5** | **ENSG00000165300** | **0.01600506** |
| HHEX |  | ENSG00000152804 | 0.01596346 |
| FANCE |  | ENSG00000112039 | 0.01591581 |
| RBM8A |  | ENSG00000131795 | 0.015864054 |
| ZYG11A |  | ENSG00000203995 | 0.015837664 |
| MSI1 |  | ENSG00000135097 | 0.015833167 |
| ZIC5 |  | ENSG00000139800 | 0.015814804 |
| ENSA |  | ENSG00000143420 | 0.015797047 |
| MCOLN3 |  | ENSG00000055732 | 0.015795384 |
| SEMA4D |  | ENSG00000187764 | 0.015776688 |
| MARS2 |  | ENSG00000152428 | 0.015757736 |
| GALNT14 |  | ENSG00000158089 | 0.015756221 |
| HOXD13 |  | ENSG00000128714 | 0.015708175 |
| **FXYD6** | **FXYD Domain Containing Ion Transport Regulator 6** | **ENSG00000137726** | **0.015698582** |
| FAF1 |  | ENSG00000185104 | 0.015683189 |
| CDC25A |  | ENSG00000164045 | 0.01567854 |
| NDRG2 |  | ENSG00000165795 | 0.015672687 |
| FAM184A |  | ENSG00000111879 | 0.015658088 |
| COIL |  | ENSG00000121058 | 0.015645424 |
| **NBPF6** | *Neuroblastoma Breakpoint Family Member 6* | ENSG00000186086 | 0.015635064 |
| ORC1 |  | ENSG00000085840 | 0.015619682 |
| GPM6A |  | ENSG00000150625 | 0.015615854 |
| PNN |  | ENSG00000100941 | 0.015608638 |
| **PRAME** | **Preferentially Expressed Antigen In Melanoma** | **ENSG00000185686** | **0.015601972** |
| MANEAL |  | ENSG00000185090 | 0.015600426 |
| C6orf141 |  | ENSG00000197261 | 0.015591807 |
| GJA3 |  | ENSG00000121743 | 0.015591593 |
| CBX2 |  | ENSG00000173894 | 0.015576485 |
| PAK6 |  | ENSG00000137843 | 0.015543478 |
| EEF1A2 |  | ENSG00000101210 | 0.015521194 |
| LLGL2 |  | ENSG00000073350 | 0.015505856 |
| CEP72 |  | ENSG00000112877 | 0.015494013 |
| CXADR |  | ENSG00000154639 | 0.015492248 |
| HERC5 |  | ENSG00000138646 | 0.015484651 |
| MGAT5B |  | ENSG00000167889 | 0.015478886 |
| NEWGENE135 |  | NEWGENE135 | 0.015444656 |
| ONECUT2 |  | ENSG00000119547 | 0.01542927 |
| KIF26A |  | ENSG00000066735 | 0.015417323 |
| C11orf9 |  | ENSG00000124920 | 0.015410247 |
| SIX3 |  | ENSG00000138083 | 0.01536409 |
| CCDC3 |  | ENSG00000151468 | 0.015358422 |
| JPH3 |  | ENSG00000154118 | 0.015345322 |
| C1QL1 |  | ENSG00000131094 | 0.015329199 |
| ZIC2 |  | ENSG00000043355 | 0.015266475 |
| CEP152 |  | ENSG00000103995 | 0.015262838 |
| GKAP1 |  | ENSG00000165113 | 0.015259934 |
| SSTR2 |  | ENSG00000180616 | 0.015255518 |
| HMX2 |  | ENSG00000188816 | 0.015252992 |
| VPS72 |  | ENSG00000163159 | 0.015244472 |
| MRPS18B |  | ENSG00000204568 | 0.015237851 |
| FBXO41 |  | ENSG00000163013 | 0.015234059 |
| L2HGDH |  | ENSG00000087299 | 0.015218391 |
| GTF3C5 |  | ENSG00000148308 | 0.015214379 |
| PCYT1B |  | ENSG00000102230 | 0.015204357 |
| EYA4 |  | ENSG00000112319 | 0.01518136 |
| C1orf77 |  | ENSG00000160679 | 0.015173888 |
| HPGD |  | ENSG00000164120 | 0.015157587 |
| CDT1 |  | ENSG00000167513 | 0.015131047 |
| AL358813.2 |  | ENSG00000203815 | 0.015111677 |
| CDCA7 |  | ENSG00000144354 | 0.015111044 |
| C5orf39 |  | ENSG00000177721 | 0.015080584 |
| RP11-528L24.3 |  | ENSG00000234602 | 0.015066763 |
| PARD6B |  | ENSG00000124171 | 0.015045202 |
| USP39 |  | ENSG00000168883 | 0.015042107 |
| SF3B4 |  | ENSG00000143368 | 0.015023954 |
| RBM38 |  | ENSG00000132819 | 0.015013925 |
| RPS6KA1 |  | ENSG00000117676 | 0.015010226 |
| LYSMD2 |  | ENSG00000140280 | 0.014985726 |
| PRKAA2 |  | ENSG00000162409 | 0.014973579 |
| IQGAP2 |  | ENSG00000145703 | 0.01495951 |
| RBBP4 |  | ENSG00000162521 | 0.014947648 |
| FOXA2 |  | ENSG00000125798 | 0.014931908 |
| CHD1L |  | ENSG00000131778 | 0.014931288 |
| AURKB |  | ENSG00000178999 | 0.014928699 |
| ITPRIPL1 |  | ENSG00000198885 | 0.014922271 |
| DKC1 |  | ENSG00000130826 | 0.01491385 |
| MCM7 |  | ENSG00000166508 | 0.014913033 |
| RNF175 |  | ENSG00000145428 | 0.014905566 |
| CALML4 |  | ENSG00000129007 | 0.014905313 |
| C1orf135 |  | ENSG00000127423 | 0.014860969 |
| CCDC21 |  | ENSG00000130695 | 0.014859658 |
| NASP |  | ENSG00000132780 | 0.014852291 |
| LASS2 |  | ENSG00000143418 | 0.014844174 |
| HS6ST2 |  | ENSG00000171004 | 0.014840284 |
| PTCD3 |  | ENSG00000132300 | 0.014839647 |
| MSH6 |  | ENSG00000116062 | 0.014837861 |

The component 1 axis of principal component analysis is expanded as the order of coefficient variables in the global transcriptome analysis. Using the whole database of global transcriptomics, the principal component analysis was performed by Strand NGS software.
